# Towards a capability approach to child growth: A theoretical framework

**DOI:** 10.1111/mcn.12534

**Published:** 2017-10-20

**Authors:** Hinke Haisma, Sepideh Yousefzadeh, Pieter Boele Van Hensbroek

**Affiliations:** ^1^ Population Research Centre University of Groningen Groningen the Netherlands; ^2^ Globalisation Studies Groningen University of Groningen Groningen the Netherlands

**Keywords:** capabilities, child growth, child mortality, context, inequalities, multidisciplinary approaches

## Abstract

Child malnutrition is an important cause of under‐5 mortality and morbidity around the globe. Despite the partial success of (inter)national efforts to reduce child mortality, under‐5 mortality rates continue to be high. The multidimensional approaches of the Sustainable Development Goals may suggest new directions for rethinking strategies for reducing child mortality and malnutrition. We propose a theoretical framework for developing a “capability” approach to child growth. The current child growth monitoring practices are based on 2 assumptions: (a) that anthropometric and motor development measures are the appropriate indicators; and (b) that child growth can be assessed using a single universal standard that is applicable around the world. These practices may be further advanced by applying a capability approach to child growth, whereby growth is redefined as the achievement of certain capabilities (of society, parents, and children). This framework is similar to the multidimensional approach to societal development presented in the seminal work of Amartya Sen. To identify the dimensions of healthy child growth, we draw upon theories from the social sciences and evolutionary biology. Conceptually, we consider growth as a plural space and propose assessing growth by means of a child growth matrix in which the context is embedded in the assessment. This approach will better address the diversities and the inequalities in child growth. Such a multidimensional measure will have implications for interventions and policy, including prevention and counselling, and could have an impact on child malnutrition and mortality.

## 1 INTRODUCTION

Child malnutrition is an important underlying cause of child morbidity and mortality (Black et al., 2013). Although the current approaches to child growth have contributed to reductions in child mortality, 6 million children still die every year (You et al., 2015). The Sustainable Development Goals (SDGs) have suggested developing a multisectoral, multidimensional approach to achieving the 2030 targets (UN Department of Economic and Social Affairs, 2015). This may also open the way for a multidimensional approach to child growth, with implications for its measurement. We suggest the adoption of a capability approach to child growth and offer a theoretical framework for its development.

**Key messages**

- Child malnutrition is an important underlying cause of child morbidity and mortality.
- High levels of child mortality persist.
- We suggest adopting a multidimensional approach to child growth that uses Sen's capability approach as a conceptual framework; this entails the construction of a growth matrix in which context will be embedded in the actual growth assessment.
- Thus, we introduce the notions of rights, justice, and inequalities into the discourse on child growth.
- This will have consequences for (a) measurement and monitoring of growth of individual children, (b) training of health professionals, (c) counselling of caregivers and prevention practices, (d) development of policy and interventions, and (e) comparisons between countries.

### 1.1 Child mortality and malnutrition

Malnutrition is an important factor underlying child mortality (Black et al., 2013). In the 1970s, charts for growth monitoring were introduced as a tool to identify children at risk of malnutrition and to improve efforts to reduce child morbidity and mortality. In the era of the Millennium Development Goals (MDGs), under‐five mortality dropped by 53%, from 91 deaths per 1,000 live births in 1990 to 43 deaths per 1,000 in 2015 (You et al., 2015). However, only about one third of the countries (62/195) managed to reduce their under‐five mortality rates by two thirds or more and achieved the MDG 4 target specified in 2000. Huge disparities exist between subpopulations. For example, in Brazil, the under‐five mortality rate has declined at the country level from 61 deaths per 1,000 live births in 1990 to 16 deaths per 1,000 in 2015, a 73% reduction. However, the rate exceeded 80 deaths per 1,000 in 32 municipalities out of the roughly 5,500 municipalities in Brazil (You et al., 2015). The Sustainable Development Goals (SDGs) were developed as a framework following the MDGs and include further aspirations to reduce child mortality around the globe. For projects under this new framework, a multistakeholder, multidimensional, and collaborative approach will be the norm (UN Department of Economic and Social Affairs, 2015).

Traditionally, the discourse on child malnutrition has been the domain of nutritionists and paediatricians, and anthropometric measures of child growth have been used to identify malnourished children. The multisectoral aspirations of the SDGs suggest that this may be an appropriate time to revisit the current approach to child growth and to work towards crafting an approach that is multidimensional, that is, an approach that takes into account contextual differences and acknowledges the importance of focusing on equality and reaching all members of society. This shift would have implications for (a) the measurement and the monitoring of the growth of individual children, (b) the training of health professionals, (c) the counselling of caregivers, (d) the development of policy and interventions aimed at preventing poor child growth, and (e) the ability to make comparisons across countries.

Such an approach requires an open mindedness towards how we think about the concept of growth. An example is how we conceptualise the sky. A sky can be looked at from different perspectives. We may look at the sky as something that tells us what the weather is like, and what we should wear. Or we may look at the sky and see its beauty and want to make photos. Astronomers look at the sky as the place where the stars are or as the start of the universe. Colour specialists look at the sky in terms of reflection of light. So although we all look at the same sky, we see something different; and if we wanted to picture the sky, we would do that all differently. Yet, how we look at growth has been dominated by nutritionists and paediatricians and as a consequence also how growth is being assessed is dominated by the biomedical paradigm. We aim to add another perspective to this biomedical one.

### 1.2 Current growth monitoring practices

Child growth has been defined by the WHO and UNICEF as “the change in weight, height, and circumference of head”; and child growth monitoring has been defined as “the process of following the growth rate of a child in comparison to a standard by periodic anthropometric measures” (UNICEF, 2008). This approach to growth monitoring dates back to the 1950s (Morley & Woodland, 1979) and reflects the biomedical perspective.

International organisations became systemically involved in growth monitoring when the WHO started recommending the use of the National Center for Health Statistics growth chart in the late 1970s. These early charts were *descriptive* in nature and were constructed from growth data from four sources in the United States. Several decades later, it was argued that there was a need for a *normative* growth curve that describes how children around the globe *should grow*. To create this curve, data were collected as part of the Multicentre Growth Reference Study that had study sites in Brazil, Ghana, India, Oman, Norway, and the United States. The subjects were selected on the basis of the following criteria: (a) they were being fed according to the WHO recommendations (i.e., 6 months of exclusive breastfeeding followed by breastfeeding with complementary foods until age two); and (b) their socio‐economic status (SES) was high (WHO, Working Group on the Growth Reference Protocol, 1998). The WHO charts monitor children's growth using a *universal standard* for all children below the age of five (de Onis, Onyango, Borghi, Garza, & Yang, 2006). The indicators used to measure growth are weight, height, body mass index (BMI), head and arm circumference, subscapular and triceps skinfolds, and motor development. The charts are accompanied by a training course for health professionals instructing them on how growth patterns should be interpreted and translated into advice to caregivers (Onyango & De Onis, 2008).

Two basic assumptions underlie the growth charts:

1. Anthropometric and motor development measures are the appropriate indicators for growth assessment; and
2. Child growth can be assessed using a single universal growth standard that is applicable to all children around the world.

Thus, growth is assessed as a *monodimensional outcome*.

The idea of measuring child growth with biometric methods is rooted in a biomedical paradigm. Moreover, it is based on the assumption that there is a “naturally developing child” 1“Childhood” is generally defined as “the period between birth and 8 years of age” (UNICEF, 2008), although different societies and cultures may adopt different cut‐off points., that is, (a) that a child is a natural phenomenon (rather than a social or a cultural phenomenon); and (b) that maturation is a universal, biologically conditioned process (James, Jenks, & Prout, 1998; James & Prout, 1997). By contrast, a multidimensional approach to child growth recognises that child growth is a socially constructed, as well as a natural phenomenon. This assumption casts a very different light on the question of "how to define healthy child growth" (Haisma & Yousefzadeh, work in progress). Applying a constructivist approach to child growth means that growth is examined and monitored in the context of each child's cultural and social conditions. Cases of poor child growth that appear similar from a biological perspective may arise in very different situations and have a wide range of causes. For example, a malnourished child could be a boy who lives in a low SES household in a welfare state, and who thus has access to market goods, high levels of mobility, and publicly provided child care; or a girl in a refugee camp with a single parent who is surviving on humanitarian services and handouts. Although both children are malnourished, their experiences of malnutrition differ, and advising and intervening in these cases require us to unpack their different realities. We propose adding these cultural and social conditions as dimensions to the growth assessment process. Thus, the goal is to measure the weight and height of each child while also collecting information on other dimensions that will help us determine how and at what level we should intervene to improve the capabilities needed to achieve healthy child growth.

**Figure 1:**
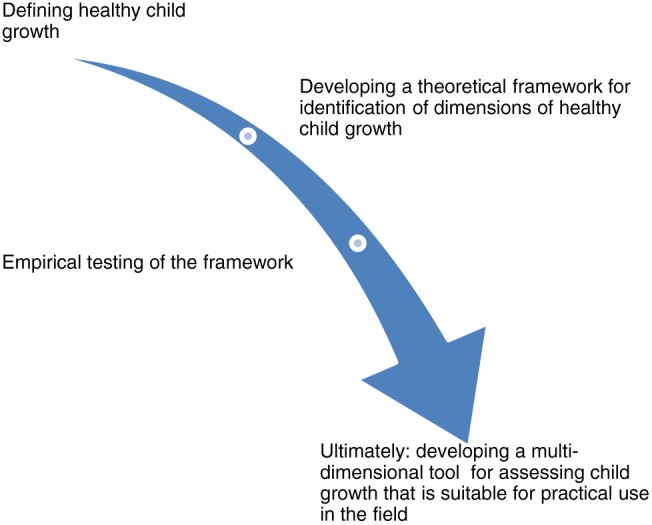
Towards a capability approach to child growth: Steps to be taken

In this paper, we introduce a theoretical framework by applying theories from different disciplines that could provide us with new directions for further developing and operationalising a multidimensional approach to child growth. This approach could evolve into a new paradigm of child growth measurement that sees context as embedded in growth assessment. In describing this framework, the differences and the inequalities in child growth will become clearer, and interventions can be better targeted towards ameliorating such inequalities. The steps that must be taken to advance this paradigm can be illustrated as in Figure 1.

## 2 DEFINING MULTIDIMENSIONAL CHILD GROWTH

Over the years, several frameworks have been developed within the biomedical paradigm for understanding determinants of child health. Mosley and Chen (1984) developed an analytical framework for child survival that includes determinants identified by both the biological and the social sciences. Their model includes five groups of proximate determinants of child survival: maternal factors, environmental contamination, nutritional deficiencies, injury, and personal illness control. They further argued that socio‐economic determinants influence child survival indirectly through proximate determinants. In 1990, UNICEF introduced a conceptual framework for the causes of child malnutrition that identified three categories of determinants (UNICEF, 1990): the basic determinants at the societal level, the underlying determinants at the household and community levels, and the immediate causes at the individual level.

These frameworks have been widely applied in multivariate data analysis (as explanatory variables) and have been seen as relevant for elaborating a hierarchy of the variables, and for subsequently understanding the roles of these variables in the causal chain (Victora, Huttly, Fuchs, & Olinto, 1997). In addition, the UNICEF framework has been instrumental in the development of interventions aimed at improving child nutrition using multisectoral approaches. More recently, Stewart et al. (2013) presented a conceptual framework for stunted growth and development that builds on the UNICEF framework by emphasising the importance of the societal and community contexts that contribute to childhood stunting. Like these authors, we acknowledge the importance of context and the need for integrated programmes. However, there is a fundamental difference between their framework and our proposed approach. Whereas Stewart et al. (2013) employed a monodimensional concept of growth and used contextual variables as *explanatory* variables, we take this approach a step further by including contextual differences within the aggregated multidimensional outcome measure of child growth; as will become clear in the course of the paper. Thus, we are assessing growth as a plural space in which context is embedded in the growth assessment, rather than addressing the context through a set of external variables. This distinction has important consequences for monitoring practices and addressing issues of inequality.

Defining the dimensions to be included in a multidimensional approach to child growth is not an easy task. We propose several ways to identify these dimensions: (a) developing a theoretical framework to elaborate the various dimensions (this paper); (2) giving voice to the people in the community by applying an emic 2The term “emic” refers to the perspective of the insider: for child growth, this means the perspective of the caregivers and local health professionals. “Emic” is contrasted with “etic” that refers to the perspective of the observer, such as a researcher or an international expert. Ethnographies apply an emic approach to understanding constructs of behaviour. approach (Hennink, Hutter, & Bailey, 2011; Pelto, 2015), which could uncover additional dimensions of child growth that have not been considered in current academic theorising; and 3) meeting with multiple stakeholders, including scientists from various disciplines, international organisations (WHO and UNICEF), health professionals, and caregivers.

## 3 TOWARDS A CAPABILITY APPROACH TO CHILD GROWTH

**Figure 2:**
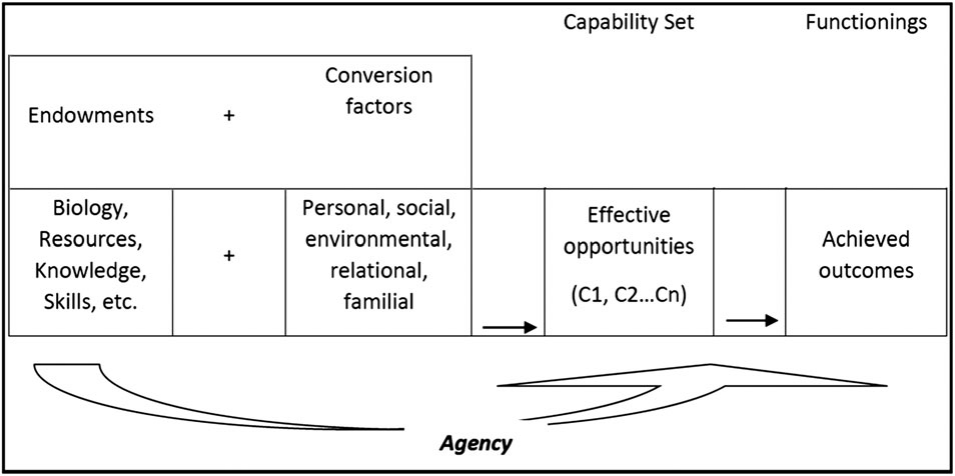
The constitutive elements of the capability approach (Chiappero‐Martinetti & Venkatapuram, 2014)

The shift towards a multidimensional approach to child growth that we propose is modelled on Nobel laureate Amartya Sen's proposal to measure societal development not just based on economic growth, but based on other dimensions, such as education and life expectancy (Sen, 1999; Sen, 2005). In a similar vein, we suggest that the definition of child growth should not be restricted to physical growth but should rather be expanded to incorporate parental, societal, and other dimensions. The capability approach has been widely applied in studies of child poverty (Biggeri & Mehrotra, 2011; Schweiger & Graf, 2015; Yousefzadeh Daal Faghati & Gossmann, 2016), well‐being (Biggeri, Ballet, & Comim, 2011; Fegter & Richter, 2014), and education (Biggeri & Santi, 2012). In child growth, however, the capability approach has not been applied, and this paper aims to explore how a capability approach to child growth could be developed. The constructive elements of the capability approach are presented in Figure 2 (Chiappero‐Martinetti & Venkatapuram, 2014), and each of these elements will be discussed in relation to child growth in the sections below.

### 3.1 Capabilities

Such a “capability approach” helps identifying different dimensions and the key capabilities that constitute healthy growth. These capabilities can refer to the child's, the caregiver's, or the society's opportunities to achieve healthy growth. Capabilities are valuable *doings or beings,* reflecting individual freedom and choices; they constitute the real, available opportunities from which people can choose. The achieved doings or beings (the functionings) follow from the capabilities, reflecting the extent to which the opportunities have been transformed into achieved outcomes.

**Figure 3:**
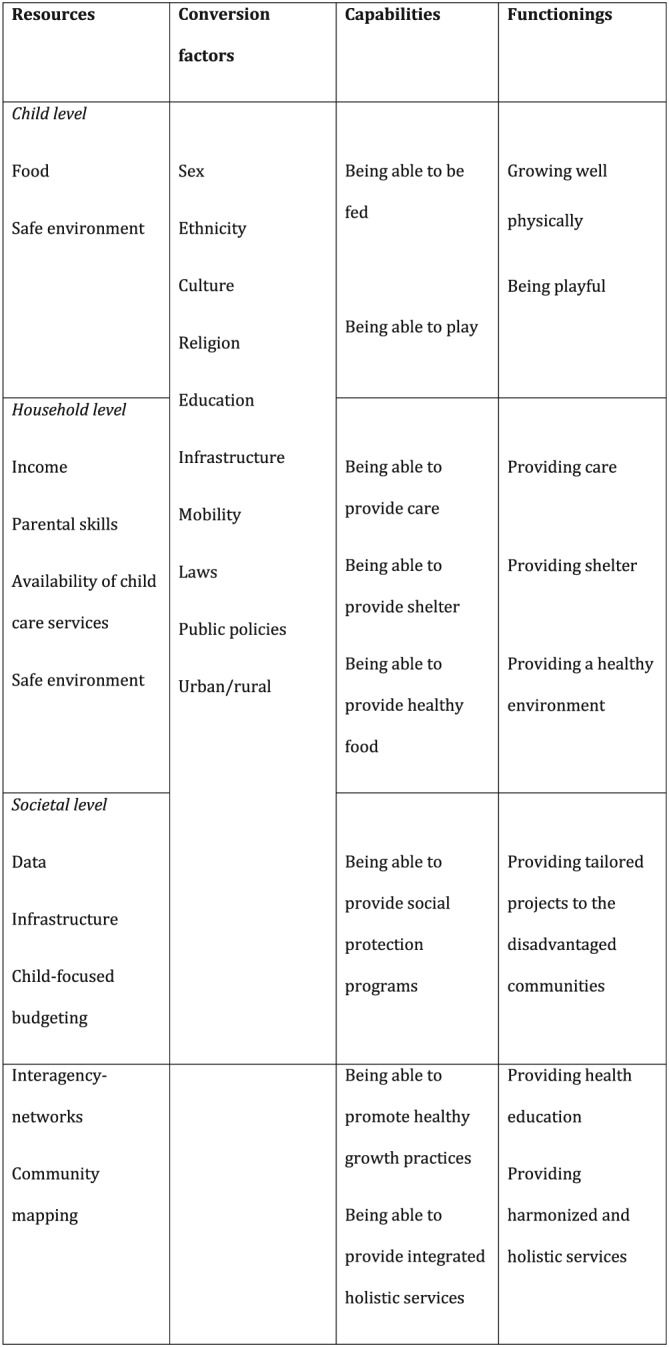
Potential capability set of child growth, including dimensions at the child, household, and the societal level. Physical growth is embedded into a multidimensional vector that includes context

The implication of this approach is that growth can be defined as *the achievement of certain capabilities*. Such capabilities would include physical growth, but the physical dimension would be part of an aggregated measure that would also include other interdependent dimensions at the child, household, and societal level. As an example, we present a potential capability set of child growth in Figure 3. Physical growth is one dimension of the aggregated growth matrix at the child level; a capability for this dimension could be “being able to be adequately fed,” with weight and height being possible indicators. Other dimensions could refer to capabilities at the household level, such as “being able to provide care” or “being able to provide shelter.” At the societal level, we could include capabilities such as “providing health education.” The advantage of including other dimensions at their respective levels into the actual growth assessment is that it immediately draws the attention to the various levels of intervention and the corresponding agency.

### 3.2 Resources and conversion factors

Figure 3 further illustrates how resources and conversion factors shape capabilities. Knowledge of these factors is essential when analysing and seeking to determine the extent to which the capabilities can be operationalised and transformed into achieved outcomes or functionings. In order to understand a situation by taking into account the concrete context, we need to map more than just the actor's material conditions; that is, resources such as family context, income, food, and school. We should also map the factors conditioning whether, and in what quantity and form, these resources are actually available to the actor (child, caregiver, or society). The combination of these resources and these factors (“conversion factors”) condition the capabilities of the actor. Examples of conversion factors are age, sex, gender, caste, ethnic group, religion, and public policies. The following example illustrates the importance of endowments and conversion factors to the capability set. If in a poor family, the daughter is the last to eat, then, even though food is available in the family, the “capability to feed or provide food" may not be fulfilled for all members equally (household level), and the “capability to grow well” may not be flourished at the (girl) child level. Being a girl “converses” this child's opportunities. Similarly, if abundant food is available, the actual capability to convert this food into healthy physical growth (e.g., avoiding diabetes in later life) may depend on the macro bio‐histories in which the child is partaking, as the example of India in the section on life history shows. The clear implication is that public health interventions must target those capability sets in order to maximise the “capability to be well‐nourished”.

### 3.3 Human choice and agency

Another aspect of the capability approach that is relevant to child growth is that of human choice and agency. When Amartya Sen moved beyond the so‐called basic needs approach to propose the capability approach in human development, he recognised that there is no standard and neutral set of needs or development aims that can be objectively fixed (Sen, 2003). Rather, the outcomes achieved are conditioned by the choices and freedom of actors. Individuals and societies choose from a capability set that they have in order to achieve the outcomes they value. When considering child growth, the corollary of moving from a basic needs approach to a capability approach involves respecting the possible differences in opinion about what “healthy” child growth is. From their available capabilities (the “capability set”), the various actors make the choices they believe will help them achieve the life course they value. As these choices are reflected in the achieved outcomes or functionings, they ultimately affect the aggregated measure of multidimensional child growth.

Ideally, people have the freedom to make choices. In the field of child growth, the choices of several actors are taken into account, including those of the child, the child's parents, other caregivers, and health professionals (Biggeri, Bellanca, Bonfanti, & Tanzj, 2011). For example, an actor may not be able to freely choose how the child is fed. While health promotion programmes advocate breastfeeding, industries advertise artificial feeding methods. Moreover, an actor's peers may or may not support breastfeeding. Thus, choice becomes a complex issue, and these complexities should be taken into account when studying capabilities of child growth.

The choice aspect of the application of a capability approach to child growth also leaves space for unhealthy choices that reflect people's values and norms. Although this may seem unwise, giving voice to people can facilitate a communication process that can generate information that may prove useful in developing public health interventions. If these interventions are embedded in people's culture and reality, they are more likely to be more effective. For example, it is known that overweight in children is a problem in a particular area in the province of Groningen in the Netherlands. However, the people who live in this region report being annoyed by public health interventions that address this issue, as they do not perceive overweight as a problem. They may choose to eat unhealthy snacks and to offer their children soft drinks for breakfast. Promoting a healthy lifestyle without understanding how food choices are made is not likely to result in desired growth outcomes. Including contextual dimensions—such as the historical background of the area and how the population perceive authorities trying to intervene in their way of life—may improve our understanding of the underlying issues and result in the development of interventions that are tailored to the needs of that particular population (Visser, 2016). As we suggested above, this approach would also need to include an analysis of the powerful interest groups that undermine individual agency, such as the food industry.

### 3.4 A multidimensional child growth index

A final parallel to Sen's approach could be the Human Development Index, which is published annually by the United Nations Development Programme. It elaborates Sen's approach into a practical multidimensional measure of development. Ultimately and after thoughtful consultation with various stakeholders, a final outcome of our effort to develop a capability approach to child growth could be to develop a Multidimensional Child Growth Index.

Thus, taking our inspiration from Sen's capability approach, we believe that addressing the issue of child growth will involve four additional steps: (a) identifying a multidimensional capability set for child growth; (b) categorising the capabilities and their resources and conversion factors at the child, household, and societal levels; (c) acknowledging the roles played by choice and agency in the achieved outcomes at all levels; and (d) generating an aggregate (i.e., multidimensional) measure of child growth.

Such a profound paradigm shift will not be achieved overnight and can only be done in thorough consultation with the relevant stakeholders. Although few members of the academic community who focus on the capability approach have conducted research on healthy child growth, this approach could prove to be useful for elaborating relevant multidimensional measures. Recently, Subramanian, Mejía‐Guevara, and Krishna (2016) suggested that the capability approach might be applied to public health issues such as child malnutrition by shifting the focus of such research to examining basic human capabilities and freedoms.

## 4 IDENTIFYING MULTIPLE DIMENSIONS OF HEALTHY CHILD GROWTH

The capability approach provides the backbone for our multidimensional approach to child growth. In identifying the dimensions, we draw upon theories and approaches from different disciplines. The capability approach is strongly rooted in a human rights‐based approach (Sen, 2005). Applying this approach is thus the first step towards identifying the dimensions of child growth. Next, we draw upon theories from demography and biology, for example, nutrition transition theory, parent–offspring conflict theory, and life history theory. These theories provide insight into how a child's place of birth or residence influence her growth trajectory; for example, a child may inherit a growth trajectory that is specific to the country where she lives that may reflect either a genotype or intergenerational nutritional or non‐nutritional factors. The nutrition transition theory provides a macro‐level nutritional perspective on child growth, whereas evolutionary life history theory and parent–offspring conflict theory provide an evolutionary biological perspective on child growth.

### 4.1 The Rights of the Child

The various international legal declarations on the rights of children enlarge the scope of considerations that come into play when analysing child health and child growth. Reference to a child's right to development was explicitly made in the Geneva Declaration of the Rights of the Child of the League of Nations (Jebb, 1923): “The child must be given the means needed for its *normal* development, both materially and spiritually.” The UN Declaration on the Rights of the Child of United Nations General Assembly (1959) included the following statement: “The child shall enjoy special protection and shall be given opportunities to develop in a healthy and normal manner, and in conditions of freedom and dignity”. Finally, the Convention on the Rights of the Child established “the best interests of the child” (Article 3) as a guiding principle (United Nations General Assembly, 1989). These discourses on the rights of the child can be taken as the basis for the development of a multidimensional approach to child growth. The rights‐based approach will help us identify capabilities relevant to child growth. Biggeri and Mehrotra (2011) identified categories of capabilities that are relevant for children based on the Rights of the Child: life and physical health, love and care, mental well‐being, bodily integrity and safety, social relations, participation, education, freedom from economic and noneconomic exploitation, shelter and environment, leisure activities, respect, religion and identity, time autonomy, and mobility. We will refer to this list of categories in identifying the dimensions of child growth.

### 4.2 Nutrition transition theory

The nutrition transition theory proposed by Popkin (1994) describes the changes in dietary and physical activity patterns of populations over time, which have resulted in a shift away from undernutrition and towards over‐nutrition, as well as a shift away from health risks associated with infectious diseases and towards health risks associated with nutrition‐related, non‐communicable diseases such as diabetes and cardiovascular diseases (Popkin, 2002). These changes in dietary patterns are paralleled by changes in lifestyle and health status, as well as by major demographic and socioeconomic changes, such as globalisation, urbanisation, industrialisation, and migration (Popkin & Gordon‐Larsen, 2004). This theory helps us better understand how population‐level food intake and the subsequent health outcomes are influenced by their macro‐level context.

Popkin's more recent work (Popkin, Adair, & Ng, 2012) has pointed to the link between nutrition transition theory and biology. This is an important step in the development of the theory, as such a biocultural approach recognises the important role that population‐level biological processes play in nutrition and health outcomes (Himmelgreen, Cantor, Arias, & Romero Daza, 2014). The biocultural approach helps to explain why the physiological effects of nutritional status differ across countries. These differences can be illustrated by examples from India and Brazil that are currently undergoing the nutrition transition (moving from Stages 3 to 4). Although both countries are considered upcoming economies, they have different biological histories (see also the section on evolutionary biology below) and therefore different aetiologies of overweight and the associated health effects.

In India, the prevalence of low birthweight is very high. These low birthweight babies typically retain fat at the expense of lean mass (the “thin‐fat” babies; Yajnik et al., 2003). This condition predisposes these babies to develop diabetes later in life (Harder, Rodekamp, Schellong, Dudenhausen, & Plagemann, 2007; Lindsay et al., 2000). It is not clear what advice regarding weight gain is appropriate for these children (Fall et al., 2008; Jain & Singhal, 2012). Rapid weight gain in early childhood has been found to increase the risk of developing the metabolic syndrome 3Metabolic syndrome refers to a cluster of risk factors for developing cardiovascular disease, namely, diabetes and prediabetes, abdominal obesity, high cholesterol, and high blood pressure. in low birthweight children (Singhal & Lucas, 2004). However, this same rapid weight gain may increase a child's chances of survival in the short‐term. For example, a study in Brazil found that children who gained weight rapidly had reduced hospitalisation rates (Victora, Barros, Horta, & Martorell, 2001). Thus, in the Indian context—which is characterised by a high prevalence of low birthweight babies, high rates of child morbidity and mortality, and a high prevalence of diabetes—a strong focus on high scores for anthropometric parameters, such as those in the growth charts, may lead to increased vulnerability to disease in later life (see the section below).

Brazil is another country where the prevalence of obesity is increasing dramatically (Ministério da Saúde, 2010). In this country, however, food patterns and associated inequalities in health outcomes are strongly linked to socio‐economic position. Post, Victora, Barros, Horta, and Guimaraes (1996) showed that as the southern Brazilian city of Pelotas underwent the nutrition transition between 1982 and 1993; overweight went from being a problem among high SES population groups to being a problem among low SES population groups. Otero, Pechlaner, and Can Gürcan (2015) suggested that this shift can be partly explained by the influence of neo‐liberalism and related changes in food patterns, as traditional foods were replaced with processed foods, including soft drinks. Popkin et al. (2012) countered that the association between SES and the prevalence of overweight is less clear‐cut and may be changing over time. The Brazilian situation suggests that an approach to healthy child growth should be sensitive to context and especially to socio‐economic position.

In sum, low birthweight is a major determinant of later health outcomes associated with metabolic syndrome in India, whereas in Brazil the primary cause of overweight and associated diseases appears to be a change in food patterns (Agras, Kraemer, Berkowitz, & Hammer, 1990). These contrasting findings fit a “capacity‐load” model of cardio‐metabolic risk, in which growth patterns are assumed to have two different associations with chronic disease in adult life, that is, poor growth in early life reduces the “metabolic capacity” for homeostasis, whereas excess growth (obesity) represents a metabolic load, challenging homeostasis (J. C. Wells, 2011).

In addition, the nutrition transition theory can help us better understand the differences between population subgroups, which is particularly important given that the current growth curves are based on infants from high SES populations (WHO, Working Group on the Growth Reference Protocol, 1998). Measurement standards on the basis of high SES populations may not apply in all contexts, as our own research in Brazil on the influence of SES on energy utilisation in infants has shown (Haisma et al., 2006). By measuring total daily energy expenditure using stable isotope techniques, we found that low SES infants used more energy than high SES infants. Although this finding confirmed our initial hypothesis that children with a low SES would use more energy, this gap between SES groups was not attributable to differences in morbidity, but rather to differences in activity levels. High SES infants tended to have a higher probability to be overweight because they were using less energy. These results suggest that in countries undergoing the nutrition transition, it is not always obvious that socio‐economic group should be considered normative in terms of metabolism, and that the SES group that is chosen as representing the norm has implications for the measurement of both physical growth patterns and behavioural patterns.

In the context of the capability approach, the nutrition transition theory can help us identify the dimensions that might be included in a capability set of child growth. The dimensions could be identified from the health outcomes of the transition (e.g., being able to access health information, being able to be adequately fed, or being able to be physically active). The indicators could include the prevalence of low birthweight or the prevalence of chronic diseases.

### 4.3 Parent–offspring conflict theory and life history theory

Although the nutrition transition theory is helpful for explaining physical growth patterns at the population level, it lacks specificity and sensitivity at the individual level. People migrate between countries at different stages in the nutrition transition or move from a situation of scarcity to a situation of affluence within a particular population (or vice versa) in space and time. Theories from evolutionary biology, such as the parent–offspring conflict theory and the life history theory, help us better understand individual growth trajectories and how they vary in association with local and parental factors. In the current institutional normative standards for physical growth and interventions based on these standards, this evolutionary component is largely ignored.

The parent–offspring conflict theory (Trivers, 1974; J. C. Wells, 2003) assumes that parents make “decisions” about feeding their offspring on the basis of maximising their reproductive fitness (defined by the number of offspring successfully raised). Although some of these decisions may be conscious, most are operationalised through physiology and represent mechanisms of plasticity that evolved through natural selection.

This theory implies that what is optimal for the mother's fitness is not necessarily optimal for the offspring. From this perspective, the optimal duration of breastfeeding for maximising a mother's reproductive fitness 4a. The genetic contribution of an individual to the next generation's gene pool relative to the average for the population, usually measured by the number of offspring or close kin who survive to reproductive age; b. the ability of a population to maintain or increase its numbers in succeeding generations depends, among other factors, on the child's sex, birth order, and birthweight; and on whether the mother has a job. Wander and Mattison's (2013) study on the evolutionary ecology of early weaning in Kilimanjaro, Tanzania, provides evidence of the importance of such context‐specific metabolic, energetic, and opportunity costs of breastfeeding and explains why human mothers often refrain from or discontinue breastfeeding. They found that (a) high SES females and low SES males were more likely to be weaned early; (b) higher parity was associated with prolonged breastfeeding; and (c) higher birthweight children were, on average, breastfed longer. These examples show how context affects maternal choices regarding infant feeding. The parent–offspring conflict theory helps to explain other phenomena as well, such as why interventions that provide additional protein and energy to the mother during pregnancy tend to have a relatively modest impact on foetal growth (Ceesay et al., 1997), and may have greater effects on maternal fertility (Gibson & Mace, 2006). Rather than being invested in the growth of the offspring, the additional energy may be retained by the mother, enabling her to produce the next offspring faster (J. C. Wells, 2003; J. C. K. Wells, Nesse, Sear, Johnstone, & Stearns, 2017).

Building on the life history theory (Stearns, 1992) and the concept of embodied capital (Kaplan, Lancaster, & Robson, 2003), Wells has suggested that “maternal capital, defined as phenotypic resources enabling investment in the offspring, allows effective buffering of the offspring from nutritional perturbations, thereby setting the trend or norm for the offspring's growth and health trajectory” (J. C. Wells, 2010b). The developmental trajectory of each offspring is thus sensitive to the amount of maternal capital available during early windows of plasticity. The offspring can respond strategically and adaptively across the life course, but only within the context of this initial maternal influence on growth. For example, among South Asian women, lower investments of energy by the mother resulted in the offspring not only having a lower birthweight but also a more rapid maturation process and a higher level of body fat, a lower height, and higher blood pressure in adulthood (J. C. K. Wells, Yao, Williams, & Gayner, 2016). Population differences in physique are developed across multiple generations, either through genetic adaptation or trans‐generational plasticity, and will continue to shape growth in future generations.

In particular, intergenerational trends in birthweight appear to be negligible, whereas secular trends in adult height and in adult BMI are pronounced (J. C. K. Wells, 2016; J. C. Wells & Stock, 2011). Rapid increases in BMI in populations with a high prevalence of undernutrition can therefore be expressed as excessive “metabolic load” that can overwhelm “metabolic capacity” and exacerbate cardiovascular risk. This pattern is reflected in the increased degenerative disease risks in populations undergoing rapid urbanisation (see the example of India above; J. C. K. Wells, 2010a; J. C. K. Wells, 2016).

This raises the key issue of whether growth is a transgenerational process that has a limited ability to “improve” within any given generation (J. C. Wells & Stock, 2011). Although foetal and infant growth are associated with long‐term benefits in terms of lean mass, rapid childhood weight gain primarily affects adiposity. Paradoxically, however, such rapid weight gain is favoured by selection in food‐insecure populations with high mortality rates, as weight gain promotes reproduction before death, and many individuals do not pay the cardio‐metabolic costs as they do not survive long enough to develop the chronic diseases associated with old age (Walker et al., 2006). Clearly, life history theory can provide important insights into a child's biological historical context that are relevant to counselling practices and intervention programmes. How many generations will it take for chronically undernourished populations to attain the physical growth patterns of the high‐SES populations upon which the WHO growth charts are based? Moreover, until these standards are attained, how should growth and development be assessed in low‐SES populations? Could we identify dimensions of child growth that would enrich our current assessments and allow us to adequately address these questions?

The above‐mentioned theories from evolutionary biology both point towards dimensions of growth that are not currently represented in growth measurement practices. Parent–offspring conflict theory is suggestive of dimensions of child growth that are related to parental care and investments in offspring. Among the capabilities that might be associated with this theory are "being able to provide good care" or "being able to receive good care". Thus, the indicators might include parenting skills, parental employment, number of siblings, order of birth, and type of feeding. Meanwhile, life history theory is linked to nutrition transition theory but involves its more physiological components. Among the capabilities that might be associated with this theory are "being able to build up body mass" and "being able to be fed". The indicators might include maternal and child body composition, energy intake, and food availability.

Thus, nutrition transition theory, parent–offspring conflict theory, and life history theory provide us with opportunities for identifying dimensions that could be included in a capability approach to child growth. Including these dimensions in the child growth matrix will enable us to better assess potential environmental challenges and children's biological histories and will facilitate the development of interventions and policies that are adapted to each child's specific reality at the societal, the household, and the individual level.

## 5 CONCLUSIONS

This paper aims to open the debate on a multidimensional approach for measuring child growth, while at the same time suggesting elements for the construction of a conceptual framework for its further development. The current discourse on healthy child growth is rooted in a biomedical paradigm that focuses on anthropometric and motor development measures of child growth only, on the basis of the assumption that growth is a purely natural and universal process. Although current growth monitoring practices have played an important role in reducing child mortality and morbidity, we argue that a different approach is needed to further reduce child mortality around the world and to increase the chances of achieving the SDGs 2030 targets. We propose developing a capability approach to child growth in which growth is redefined as the achievement of a multidimensional set of capabilities of healthy child growth. In such an approach, measures of physical child growth would be combined with other factors to create an aggregated measure of child growth, that is, a matrix of child growth (Yousefzadeh et al., work in progress). In identifying the dimensions of the capability approach, we have drawn upon The Rights of the Child, and on theories from evolutionary biology and the social sciences. The crucial difference between our proposed approach and other approaches to conceptualising child growth is the multidimensional nature of the growth assessment itself. Rather than considering contextual factors as possible causes of physical child growth, we embrace context as an integral part of the aggregated matrix that constitutes child growth. A capability approach to child growth allows us to examine the plurality of space and its various functionings in relation to the child and to the child's caregivers. We suggest that rather than examining anthropometric measures as “the dependent variable”, the plural space—that is, the multiple functionings that interact with each other—should be the focus of such measures. This approach can inform policy and interventions for prevention that are tailored to specific biological, socio‐economic, and cultural contexts. By allowing for adjustments of growth monitoring practices and of the counselling of caregivers based on the needs of the individual, this approach can help children and their caregivers realise their capabilities. Ultimately, this multidimensional approach could have an impact on child mortality.

## CONFLICTS OF INTEREST

The authors declare that they have no conflicts of interest.

## CONTRIBUTIONS

HH conceived the initial idea and wrote the first draft of the article. SY and PB contributed to its further conceptualisation and writing.
